# 2-(Di­phenyl­methyl­idene)-2,3-di­hydro-1*H*-inden-1-one

**DOI:** 10.1107/S1600536813018990

**Published:** 2013-07-24

**Authors:** Tao Zhang, Vilmar Bandero, Tom McCabe, Neil Frankish, Helen Sheridan

**Affiliations:** a Drug Discovery Group, School of Pharmacy and Pharmaceutical Sciences, Trinity College Dublin, Dublin 2, Ireland; b School of Chemistry, Trinity College Dublin, Dublin 2, Ireland

## Abstract

In the title mol­ecule, C_22_H_16_O, the indanone ring system is approximately planar with a dihedral angle between the fused rings of 5.13 (14)°. Two benzene rings are linked together at one side of a double bond, sitting on either side of the indanone ring system and making dihedral angles of 70.30 (12) and 44.74 (13)° with it. In the crystal, hydrogen bonding is not present, but weak C—H⋯π or π–π inter­actions occur and mol­ecules form a sheet-like structure in the *bc* plane.

## Related literature

For background to the indanone pharmacophore, its use as an organic inter­mediate and its biological activity, see: Buckle *et al.* (1973 ▶); Sheridan *et al.* (1990 ▶, 1999*a* ▶,*b* ▶, 2008 ▶, 2009*a* ▶,*b* ▶); Vacca *et al.* (1994 ▶); Schumann *et al.* (2001 ▶); Herzog *et al.* (2002 ▶); Frankish *et al.* (2004 ▶); Frankish & Sheridan (2012 ▶); Dinges *et al.* (2006 ▶); Kou *et al.* (2012 ▶); Ito *et al.* (2004 ▶); Jaki *et al.* (1999 ▶); Chanda *et al.* (2012 ▶); Chen *et al.* (2008 ▶); Rukachaisirikul *et al.* (2013 ▶); Farrell *et al.* (1996 ▶); Borbone *et al.* (2011 ▶); Fu & Wang (2008 ▶). For bond lengths and angles in related compounds, see: Ali *et al.* (2010*a* ▶,*b* ▶, 2011 ▶); Chen *et al.* (2011*a* ▶ 2011*b* ▶); Li *et al.* (2012 ▶); Lin *et al.* (2012 ▶).
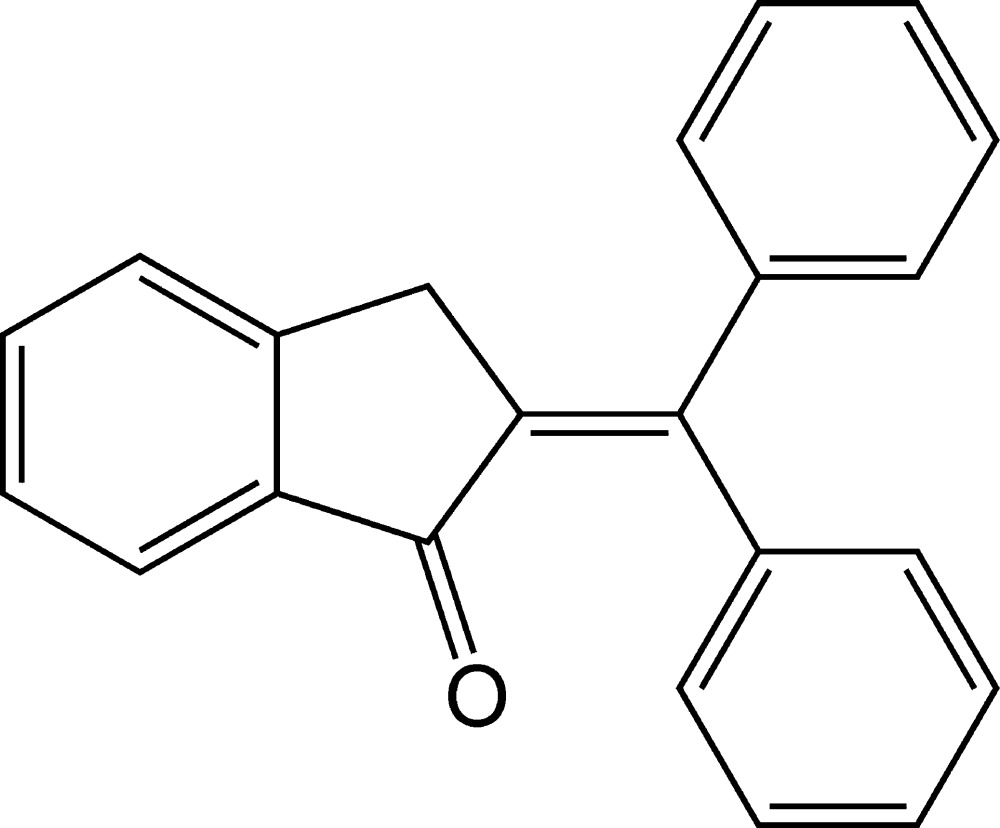


## Experimental

### 

#### Crystal data

C_22_H_16_O
*M* *_r_* = 296.35
Monoclinic, 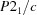

*a* = 9.1634 (18) Å
*b* = 17.570 (3) Å
*c* = 10.717 (4) Å
β = 117.89 (2)°
*V* = 1525.0 (7) Å^3^
*Z* = 4
Mo *K*α radiation
μ = 0.08 mm^−1^
*T* = 150 K
0.50 × 0.20 × 0.20 mm

#### Data collection

Rigaku Saturn 724 diffractometer
Absorption correction: multi-scan (*CrystalClear*; Rigaku, 2006 ▶) *T*_min_ = 0.763, *T*_max_ = 1.000
11746 measured reflections
2680 independent reflections
2587 reflections with *I* > 2σ(*I*)
*R*_int_ = 0.058

#### Refinement

*R*[*F* ^2^ > 2σ(*F* ^2^)] = 0.070
*wR*(*F* ^2^) = 0.145
*S* = 1.25
2680 reflections
209 parameters
H-atom parameters constrained
Δρ_max_ = 0.19 e Å^−3^
Δρ_min_ = −0.24 e Å^−3^

### 

Data collection: *CrystalClear* (Rigaku, 2006 ▶); cell refinement: *CrystalClear*; data reduction: *CrystalClear*; program(s) used to solve structure: *SHELXS97* (Sheldrick, 2008 ▶); program(s) used to refine structure: *SHELXL97* (Sheldrick, 2008 ▶); molecular graphics: *ORTEP-3 for Windows* (Farrugia, 2012 ▶) and *Mercury* (Macrae *et al.*, 2006 ▶); software used to prepare material for publication: *SHELXL97* and *Mercury* (Macrae *et al.*, 2006 ▶).

## Supplementary Material

Crystal structure: contains datablock(s) I, New_Global_Publ_Block. DOI: 10.1107/S1600536813018990/gg2117sup1.cif

Structure factors: contains datablock(s) I. DOI: 10.1107/S1600536813018990/gg2117Isup2.hkl

Supplementary material file. DOI: 10.1107/S1600536813018990/gg2117Isup3.cml

Additional supplementary materials:  crystallographic information; 3D view; checkCIF report

## Figures and Tables

**Table 1:** Hydrogen-bond geometry (Å, °) *Cg*1, *Cg*2 and *Cg*4 are the centroids of the C14–C16/C21/C22, C1–C6 and C16–C21 rings, respectively.

*D*—H⋯*A*	*D*—H	H⋯*A*	*D*⋯*A*	*D*—H⋯*A*
C1—H1⋯*Cg*1^i^	0.93	2.91	3.763 (3)	153
C11—H11⋯*Cg*2^ii^	0.93	2.99	3.712 (3)	136
C15—H15*B*⋯*Cg*4^iii^	0.97	2.92	3.640 (3)	132

Symmetry codes: (i) 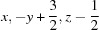; (ii) 
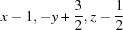
; (iii) 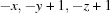.

**Fig. 1.**
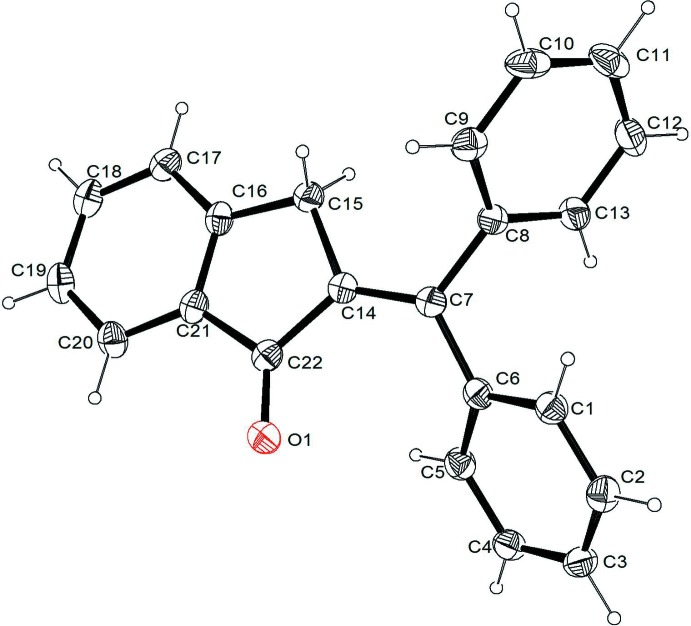
The molecule structure of the titled compound with the atom numbering scheme. Displacement ellipsoids are shown at the 50% probability level. H atoms are presented as small spheres of arbitrary radius.

**Fig. 2.**
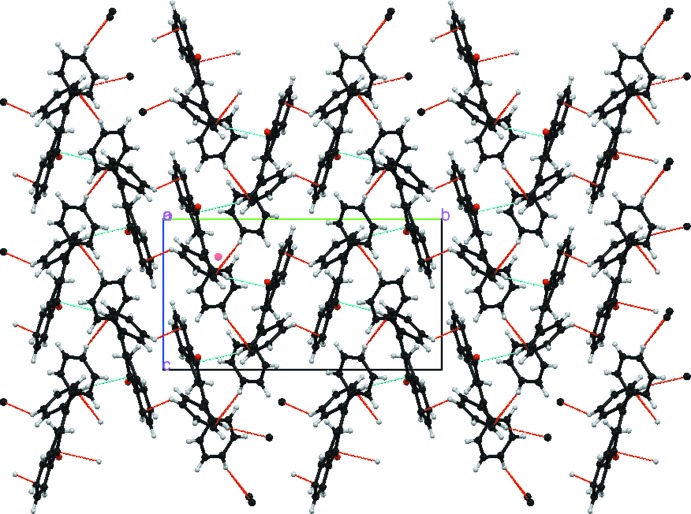
a. The molecular packing, viewed along the a axis.

**Fig. 3.**
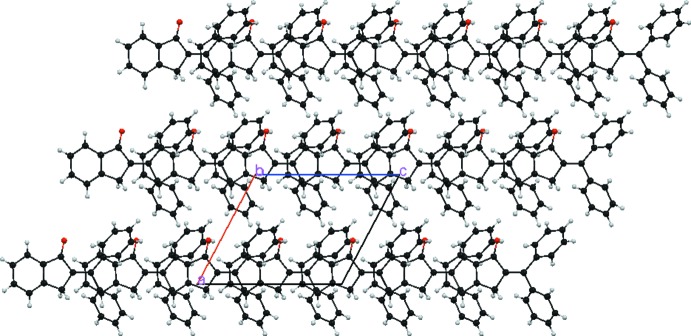
b. The molecular packing, viewed along the b axis.

## supplementary crystallographic information

### Comment

The indanone scaffold has been widely observed in the natural world (Jaki *et al.*, 1999; Ito *et al.*, 2004; Chen *et al.*, 2008; Chanda *et al.*, 2012; Rukachaisirikul *et al.*, 2013). As organic intermediates, many indanone derivatives are used during chemical synthesis (Sheridan *et al.*, 1990; Farrell *et al.*, 1996; Sheridan *et al.*, 1999*a*,*b*; Frankish *et al.*, 2004; Borbone *et al.*, 2011; Fu & Wang, 2008). Many studies show indanone pharmacophore is associated with a wide variety of biological properties such as: KDR kinase inhibition, mast cell stabilization, smooth muscle relaxation, antioxidation, and is used to target diseases such as cancer and Alzheimer's disease (Schumann *et al.*, 2001; Herzog *et al.*, 2002; Dinges *et al.*, 2006; Sheridan *et al.*, 2009*a*,*b*; Kou *et al.*, 2012).

The asymmetric unit of the title molecule (I) is shown in Figure 1. It crystallizes in the non-chiral, monoclinic space group P2_1_/c. The two benzene rings, C8—C13 and C1—C6 in the molecule lie above and below the C16—C21 plane, with the dihedral angles 70.30 (12)° and 44.74 (13)°, respectively. The torsion angles of these two benzene groups are [C14—C7—C8—C9] = 56.4 (3)° and [C14—C7—C6—C5] = 36.5 (4)°. The rest of the molecule is essentially planar. The indanone fraction shows the normal values for this type of molecules (Ali *et al.*, 2010*a*,*b*; Ali *et al.*, 2011; Chen *et al.*, 2011*a*,*b*; Li *et al.*, 2012; Lin *et al.*, 2012), with the C20—C21—C16—C15 bond angle being 176.3 (2)° and the bond length of benzylic carbonyl functionality (C22—O1) 1.231 (3) Å. The double bond (C14=C7) is located at *alpha* position to the carbonyl group of the indanone ring, with the bond length being 1.362 (4) Å. The geometry around quaternary C7 can be considered as a planar triangle: C14—C7—C8 = 119.0 (2)°, C14—C7—C6 = 126.2 (3)° and C6—C7—C8 = 114.6 (2)°. The packing diagrams of the molecular structure are presented in Figure 2. Weak intermolecular C—H···π and π-π interactions are observed in Figure 2a, which seems to be very effective in the stabilization of the crystal structure. Figure 2b shows that the molecules are separated by forming a sheet-like structure in the *bc*-plane when viewed along the crystallographic *b*-axis. It is suggested that weak Van der Vaals force or electrostatic interaction could be contributed to the linkage of the sheets.

### Experimental

To a stirred solution of 2-((2-hydroxyethoxy)diphenylmethyl)-2,3-dihydroinden-1-one (2.23 mmol) in methanol/DCM (12 ml, *v:v*, 3:1) was added trifluoromethanesulfonic acid (0.2 ml). The reaction was stirred at reflux for one hour, after which time the reaction was quenched by the addition of 2*M* NaOH aq. solution (20 ml) and the product was extracted with DCM (3 *x* 25 ml). The combined organic extracts were dried over magnesium sulfate, filtered and concentrated *in vacuo*, and the residue was purified by flash column chromatography on silica gel 230–400mesh (eluent: hexane: ethyl acetate, 10:1). All homogenous fractions were collected and the solvent removed *in vacuo* to afford titled crossed aldol condensed compound (94%) as yellow solid. Crystals suitable for X-ray diffraction were obtained after 7 days of slow evaporation of an ethyl acetate solution.

### Refinement

All H atoms were placed in geometrically idealized positions and treated using the riding model, with C—H = 0.93–0.97 Å for H atoms. *U*_iso_(H) values were set at 1.2–1.5 times *U*_eq_(C) for the H atoms in the molecule.

### Crystal data

**Table:**

C_22_H_16_O	*F*(000) = 624
*M**_r_* = 296.35	*D*_x_ = 1.291 Mg m^−^^3^
Monoclinic, *P*2_1_/*c*	Mo *K*α radiation, λ = 0.71073 Å
Hall symbol: -P 2ybc	Cell parameters from 5979 reflections
*a* = 9.1634 (18) Å	θ = 2.4–31.1°
*b* = 17.570 (3) Å	µ = 0.08 mm^−^^1^
*c* = 10.717 (4) Å	*T* = 150 K
β = 117.89 (2)°	Prism, colourless
*V* = 1525.0 (7) Å^3^	0.50 × 0.20 × 0.20 mm
*Z* = 4	

### Data collection

**Table:**

Rigaku Saturn 724 diffractometer	2587 reflections with *I* > 2σ(*I*)
Radiation source: fine-focus sealed tube	*R*_int_ = 0.058
Graphite monochromator	θ_max_ = 25.0°, θ_min_ = 2.4°
ω and φ scans	*h* = −10→10
Absorption correction: multi-scan (*CrystalClear*; Rigaku, 2006)	*k* = −20→13
*T*_min_ = 0.763, *T*_max_ = 1.000	*l* = −11→12
11746 measured reflections	4590 standard reflections every 120 reflections
2680 independent reflections	intensity decay: none

### Refinement

**Table:**

Refinement on *F*^2^	Primary atom site location: structure-invariant direct methods
Least-squares matrix: full	Secondary atom site location: difference Fourier map
*R*[*F*^2^ > 2σ(*F*^2^)] = 0.070	Hydrogen site location: inferred from neighbouring sites
*wR*(*F*^2^) = 0.145	H-atom parameters constrained
*S* = 1.25	*w* = 1/[σ^2^(*F*_o_^2^) + (0.0358*P*)^2^ + 1.5166*P*] where *P* = (*F*_o_^2^ + 2*F*_c_^2^)/3
2680 reflections	(Δ/σ)_max_ < 0.001
209 parameters	Δρ_max_ = 0.19 e Å^−^^3^
0 restraints	Δρ_min_ = −0.24 e Å^−^^3^

### Special details

**Table:**

Geometry. All e.s.d.'s (except the e.s.d. in the dihedral angle between two l.s. planes) are estimated using the full covariance matrix. The cell e.s.d.'s are taken into account individually in the estimation of e.s.d.'s in distances, angles and torsion angles; correlations between e.s.d.'s in cell parameters are only used when they are defined by crystal symmetry. An approximate (isotropic) treatment of cell e.s.d.'s is used for estimating e.s.d.'s involving l.s. planes.
Refinement. Refinement of *F*^2^ against ALL reflections. The weighted *R*-factor *wR* and goodness of fit *S* are based on *F*^2^, conventional *R*-factors *R* are based on *F*, with *F* set to zero for negative *F*^2^. The threshold expression of *F*^2^ > σ(*F*^2^) is used only for calculating *R*-factors(gt) *etc*. and is not relevant to the choice of reflections for refinement. *R*-factors based on *F*^2^ are statistically about twice as large as those based on *F*, and *R*- factors based on ALL data will be even larger.

### Fractional atomic coordinates and isotropic or equivalent isotropic displacement parameters (Å^2^)

**Table:**

	*x*	*y*	*z*	*U*_iso_*/*U*_eq_	
O1	0.3991 (2)	0.62562 (11)	0.58262 (19)	0.0266 (4)	
C1	0.2609 (3)	0.69082 (14)	0.1518 (3)	0.0216 (6)	
H1	0.1860	0.7305	0.1127	0.026*	
C2	0.3856 (3)	0.68115 (15)	0.1133 (3)	0.0241 (6)	
H2	0.3944	0.7148	0.0502	0.029*	
C3	0.4960 (3)	0.62161 (15)	0.1689 (3)	0.0256 (6)	
H3	0.5790	0.6151	0.1431	0.031*	
C4	0.4829 (3)	0.57117 (16)	0.2640 (3)	0.0253 (6)	
H4	0.5567	0.5309	0.3009	0.030*	
C5	0.3595 (3)	0.58118 (15)	0.3035 (3)	0.0230 (6)	
H5	0.3517	0.5475	0.3671	0.028*	
C6	0.2469 (3)	0.64149 (14)	0.2488 (3)	0.0200 (5)	
C7	0.1051 (3)	0.65032 (13)	0.2802 (3)	0.0192 (5)	
C8	−0.0542 (3)	0.67304 (14)	0.1560 (3)	0.0195 (5)	
C9	−0.1456 (3)	0.73524 (15)	0.1641 (3)	0.0244 (6)	
H9	−0.1056	0.7638	0.2466	0.029*	
C10	−0.2959 (3)	0.75445 (16)	0.0491 (3)	0.0306 (7)	
H10	−0.3562	0.7956	0.0551	0.037*	
C11	−0.3559 (3)	0.71206 (17)	−0.0747 (3)	0.0329 (7)	
H11	−0.4570	0.7245	−0.1510	0.040*	
C12	−0.2652 (3)	0.65121 (17)	−0.0848 (3)	0.0304 (7)	
H12	−0.3054	0.6230	−0.1677	0.036*	
C13	−0.1145 (3)	0.63269 (16)	0.0291 (3)	0.0255 (6)	
H13	−0.0528	0.5928	0.0209	0.031*	
C14	0.1063 (3)	0.63406 (14)	0.4049 (3)	0.0195 (5)	
C15	−0.0506 (3)	0.62441 (15)	0.4193 (3)	0.0227 (6)	
H15A	−0.1067	0.6728	0.4076	0.027*	
H15B	−0.1256	0.5888	0.3498	0.027*	
C16	0.0094 (3)	0.59403 (14)	0.5668 (3)	0.0208 (6)	
C17	−0.0825 (3)	0.56906 (14)	0.6321 (3)	0.0239 (6)	
H17	−0.1972	0.5682	0.5831	0.029*	
C18	0.0010 (3)	0.54542 (15)	0.7717 (3)	0.0268 (6)	
H18	−0.0591	0.5273	0.8153	0.032*	
C19	0.1738 (4)	0.54814 (15)	0.8486 (3)	0.0278 (6)	
H19	0.2266	0.5335	0.9429	0.033*	
C20	0.2664 (3)	0.57288 (14)	0.7835 (3)	0.0250 (6)	
H20	0.3810	0.5750	0.8332	0.030*	
C21	0.1817 (3)	0.59445 (14)	0.6412 (3)	0.0209 (6)	
C22	0.2509 (3)	0.61871 (14)	0.5466 (3)	0.0206 (6)	

### Atomic displacement parameters (Å^2^)

**Table:**

	*U*^11^	*U*^22^	*U*^33^	*U*^12^	*U*^13^	*U*^23^
O1	0.0225 (10)	0.0323 (11)	0.0230 (10)	−0.0011 (8)	0.0091 (8)	0.0002 (8)
C1	0.0214 (13)	0.0210 (13)	0.0214 (14)	−0.0001 (10)	0.0091 (11)	−0.0005 (10)
C2	0.0263 (14)	0.0235 (14)	0.0240 (15)	−0.0064 (11)	0.0130 (12)	−0.0018 (11)
C3	0.0200 (13)	0.0327 (15)	0.0256 (15)	−0.0035 (11)	0.0119 (12)	−0.0076 (12)
C4	0.0204 (13)	0.0296 (14)	0.0231 (15)	0.0028 (11)	0.0079 (12)	−0.0016 (11)
C5	0.0236 (13)	0.0230 (13)	0.0213 (14)	0.0013 (10)	0.0095 (12)	0.0026 (10)
C6	0.0197 (12)	0.0198 (12)	0.0184 (13)	−0.0034 (10)	0.0072 (11)	−0.0041 (10)
C7	0.0197 (13)	0.0159 (12)	0.0209 (14)	−0.0008 (10)	0.0087 (11)	−0.0019 (10)
C8	0.0191 (13)	0.0194 (12)	0.0206 (14)	−0.0003 (10)	0.0099 (11)	0.0045 (10)
C9	0.0246 (13)	0.0233 (13)	0.0289 (15)	0.0012 (11)	0.0155 (12)	0.0057 (11)
C10	0.0228 (14)	0.0286 (15)	0.0444 (19)	0.0074 (12)	0.0191 (14)	0.0168 (13)
C11	0.0203 (13)	0.0411 (17)	0.0319 (17)	0.0016 (12)	0.0077 (13)	0.0205 (14)
C12	0.0267 (15)	0.0394 (17)	0.0192 (15)	−0.0054 (12)	0.0059 (12)	0.0053 (12)
C13	0.0236 (14)	0.0301 (14)	0.0220 (15)	0.0011 (11)	0.0101 (12)	0.0041 (11)
C14	0.0211 (13)	0.0175 (12)	0.0201 (14)	−0.0005 (10)	0.0099 (11)	−0.0005 (10)
C15	0.0221 (13)	0.0251 (13)	0.0220 (14)	0.0017 (11)	0.0112 (11)	0.0021 (11)
C16	0.0280 (14)	0.0149 (12)	0.0205 (14)	−0.0005 (10)	0.0122 (12)	−0.0030 (10)
C17	0.0266 (14)	0.0209 (13)	0.0276 (15)	−0.0029 (11)	0.0156 (12)	−0.0037 (11)
C18	0.0378 (16)	0.0229 (13)	0.0257 (15)	−0.0037 (12)	0.0197 (13)	−0.0021 (11)
C19	0.0378 (16)	0.0260 (14)	0.0188 (14)	−0.0049 (12)	0.0125 (13)	−0.0010 (11)
C20	0.0280 (14)	0.0224 (13)	0.0219 (14)	−0.0072 (11)	0.0094 (12)	−0.0052 (11)
C21	0.0269 (14)	0.0158 (12)	0.0219 (14)	−0.0021 (10)	0.0130 (12)	−0.0033 (10)
C22	0.0233 (14)	0.0168 (12)	0.0229 (14)	−0.0011 (10)	0.0119 (12)	−0.0029 (10)

### Geometric parameters (Å, º)

**Table:**

O1—C22	1.231 (3)	C11—C12	1.389 (4)
C1—C2	1.395 (3)	C11—H11	0.9300
C1—C6	1.405 (3)	C12—C13	1.388 (4)
C1—H1	0.9300	C12—H12	0.9300
C2—C3	1.382 (4)	C13—H13	0.9300
C2—H2	0.9300	C14—C22	1.501 (4)
C3—C4	1.397 (4)	C14—C15	1.525 (3)
C3—H3	0.9300	C15—C16	1.508 (3)
C4—C5	1.392 (4)	C15—H15A	0.9700
C4—H4	0.9300	C15—H15B	0.9700
C5—C6	1.402 (4)	C16—C17	1.393 (4)
C5—H5	0.9300	C16—C21	1.397 (4)
C6—C7	1.495 (3)	C17—C18	1.388 (4)
C7—C14	1.362 (4)	C17—H17	0.9300
C7—C8	1.498 (3)	C18—C19	1.403 (4)
C8—C13	1.398 (4)	C18—H18	0.9300
C8—C9	1.404 (4)	C19—C20	1.396 (4)
C9—C10	1.394 (4)	C19—H19	0.9300
C9—H9	0.9300	C20—C21	1.402 (4)
C10—C11	1.391 (4)	C20—H20	0.9300
C10—H10	0.9300	C21—C22	1.488 (3)
			
C2—C1—C6	121.0 (2)	C11—C12—H12	120.1
C2—C1—H1	119.5	C12—C13—C8	121.0 (3)
C6—C1—H1	119.5	C12—C13—H13	119.5
C3—C2—C1	120.0 (2)	C8—C13—H13	119.5
C3—C2—H2	120.0	C7—C14—C22	128.9 (2)
C1—C2—H2	120.0	C7—C14—C15	123.2 (2)
C2—C3—C4	120.0 (2)	C22—C14—C15	107.8 (2)
C2—C3—H3	120.0	C16—C15—C14	104.3 (2)
C4—C3—H3	120.0	C16—C15—H15A	110.9
C5—C4—C3	120.1 (2)	C14—C15—H15A	110.9
C5—C4—H4	120.0	C16—C15—H15B	110.9
C3—C4—H4	120.0	C14—C15—H15B	110.9
C4—C5—C6	120.8 (2)	H15A—C15—H15B	108.9
C4—C5—H5	119.6	C17—C16—C21	120.1 (2)
C6—C5—H5	119.6	C17—C16—C15	128.9 (2)
C5—C6—C1	118.1 (2)	C21—C16—C15	110.9 (2)
C5—C6—C7	122.1 (2)	C18—C17—C16	118.6 (2)
C1—C6—C7	119.5 (2)	C18—C17—H17	120.7
C14—C7—C6	126.2 (2)	C16—C17—H17	120.7
C14—C7—C8	119.0 (2)	C17—C18—C19	121.6 (2)
C6—C7—C8	114.6 (2)	C17—C18—H18	119.2
C13—C8—C9	118.6 (2)	C19—C18—H18	119.2
C13—C8—C7	120.4 (2)	C20—C19—C18	120.0 (3)
C9—C8—C7	121.0 (2)	C20—C19—H19	120.0
C10—C9—C8	120.4 (3)	C18—C19—H19	120.0
C10—C9—H9	119.8	C19—C20—C21	118.1 (2)
C8—C9—H9	119.8	C19—C20—H20	120.9
C11—C10—C9	120.0 (3)	C21—C20—H20	120.9
C11—C10—H10	120.0	C16—C21—C20	121.5 (2)
C9—C10—H10	120.0	C16—C21—C22	109.9 (2)
C12—C11—C10	120.2 (3)	C20—C21—C22	128.6 (2)
C12—C11—H11	119.9	O1—C22—C21	125.0 (2)
C10—C11—H11	119.9	O1—C22—C14	128.5 (2)
C13—C12—C11	119.8 (3)	C21—C22—C14	106.5 (2)
C13—C12—H12	120.1		
			
C6—C1—C2—C3	1.1 (4)	C6—C7—C14—C15	−163.5 (2)
C1—C2—C3—C4	−0.1 (4)	C8—C7—C14—C15	10.9 (4)
C2—C3—C4—C5	−0.5 (4)	C7—C14—C15—C16	170.5 (2)
C3—C4—C5—C6	0.2 (4)	C22—C14—C15—C16	−6.8 (3)
C4—C5—C6—C1	0.7 (4)	C14—C15—C16—C17	−174.2 (2)
C4—C5—C6—C7	175.1 (2)	C14—C15—C16—C21	7.2 (3)
C2—C1—C6—C5	−1.4 (4)	C21—C16—C17—C18	0.5 (4)
C2—C1—C6—C7	−175.9 (2)	C15—C16—C17—C18	−178.1 (2)
C5—C6—C7—C14	36.5 (4)	C16—C17—C18—C19	1.8 (4)
C1—C6—C7—C14	−149.2 (3)	C17—C18—C19—C20	−2.0 (4)
C5—C6—C7—C8	−138.0 (2)	C18—C19—C20—C21	0.0 (4)
C1—C6—C7—C8	36.2 (3)	C17—C16—C21—C20	−2.5 (4)
C14—C7—C8—C13	−123.9 (3)	C15—C16—C21—C20	176.3 (2)
C6—C7—C8—C13	51.1 (3)	C17—C16—C21—C22	176.4 (2)
C14—C7—C8—C9	56.4 (3)	C15—C16—C21—C22	−4.8 (3)
C6—C7—C8—C9	−128.6 (2)	C19—C20—C21—C16	2.2 (4)
C13—C8—C9—C10	2.1 (4)	C19—C20—C21—C22	−176.5 (2)
C7—C8—C9—C10	−178.2 (2)	C16—C21—C22—O1	178.9 (2)
C8—C9—C10—C11	−0.2 (4)	C20—C21—C22—O1	−2.2 (4)
C9—C10—C11—C12	−0.9 (4)	C16—C21—C22—C14	0.2 (3)
C10—C11—C12—C13	0.1 (4)	C20—C21—C22—C14	179.1 (2)
C11—C12—C13—C8	1.9 (4)	C7—C14—C22—O1	8.6 (4)
C9—C8—C13—C12	−2.9 (4)	C15—C14—C22—O1	−174.4 (2)
C7—C8—C13—C12	177.3 (2)	C7—C14—C22—C21	−172.8 (2)
C6—C7—C14—C22	13.2 (4)	C15—C14—C22—C21	4.2 (3)
C8—C7—C14—C22	−172.5 (2)		

### Hydrogen-bond geometry (Å, º)

Cg1, Cg2 and Cg4 are the centroids of the C14–C16/C21/C22, C1–C6 and C16–C21 rings, respectively.

**Table:**

*D*—H···*A*	*D*—H	H···*A*	*D*···*A*	*D*—H···*A*
C1—H1···*Cg*1^i^	0.93	2.91	3.763 (3)	153
C11—H11···*Cg*2^ii^	0.93	2.99	3.712 (3)	136
C15—H15*B*···*Cg*4^iii^	0.97	2.92	3.640 (3)	132

Symmetry codes: (i) *x*, −*y*+3/2, *z*−1/2; (ii) *x*−1, −*y*+3/2, *z*−1/2; (iii) −*x*, −*y*+1, −*z*+1.
